# Assessment of intestinal obstruction: Clinical presentation, pathological findings and management

**DOI:** 10.6026/973206300210892

**Published:** 2025-04-30

**Authors:** Bhupesh Kushram, Archana Kori, Dinesh Kumar Thakur, Abhay Kumar, Mahendra Singh

**Affiliations:** 1 Department of General Surgery, Chhindwara Institute of Medical Sciences, Chhindwara, Madhya Pradesh, India; 2 Department of Obstetrics and Gynecology, Chhindwara Institute of Medical Sciences, Chhindwara, Madhya Pradesh, India; 3 Department of Medicine, Chhindwara Institute of Medical Sciences, Chhindwara, Madhya Pradesh, India; 4 Department of ENT, Chhindwara Institute of Medical Sciences, Chhindwara, Madhya Pradesh, India

**Keywords:** Intestinal obstruction, adhesions, bowel ischemia, surgical management, clinico-pathological correlation

## Abstract

Evaluating the clinical presentation of intestinal obstruction along with its pathological findings is crucial for accurate diagnosis and timely intervention. A total of 100 patients presenting with signs and symptoms of acute or subacute intestinal obstruction were included, and their detailed clinical histories, physical examinations, laboratory investigations, and radiological assessments were recorded. Intraoperative findings and histopathological results were correlated to identify the most common etiologies and to assess clinical outcomes. Follow-ups were done for at least six weeks post-discharge. A combined clinico-pathological approach is instrumental in effectively diagnosing and managing intestinal obstruction. Early detection, prompt surgical intervention when indicated, and appropriate postoperative care is essential to reduce morbidity and mortality.

## Background:

Worldwide, healthcare institutions encounter intestinal obstruction frequently as a surgical emergency, which causes significant death rates [1]. Medical or functional causes generate this condition when affecting either the small intestine or the large intestine simultaneously. The primary causes of mechanical blockage arise from adhesions, hernias, and tumors, as well as volvulus and functional obstructions that form due to dys-motility rather than mechanical impediments [2]. The difference between obturation forms is crucial because their management approaches and prognostic behavior differ substantially. According to various medical series worldwide, post-operative adhesions are the primary reason behind small bowel obstruction cases, which account for 40-70% of total occurrences [3]. Adult patients older than 50 years face colonic carcinoma as their primary cause of large bowel obstructions, together with neoplastic processes and volvulus events [4]. The underlying mechanism of intestinal obstruction involves the accumulation of fluid, gas, and intestinal contents proximal to the site of obstruction. This leads to bowel distension, increased intraluminal pressure, and impaired vascular supply. If untreated, it can result in bowel ischemia, necrosis, and perforation [5]. Functional obstruction, known as paralytic ileus, occurs due to neuromuscular impairment rather than a mechanical blockage, commonly seen postoperatively or in metabolic disorders [6].

Signs and symptoms of bowel obstruction appear progressively with a distended abdomen along with pain that intensifies and vomiting that is followed by obstipation; these symptoms' characteristics depend on how long and severely the obstruction exists [7]. High-grade obstructions are more likely to cause severe dehydration and electrolyte imbalances due to fluid sequestration within the bowel [8]. A delayed diagnosis presents hazardous risks because tension on the bowel tissue can lead to necrosis and sepsis as well as death. The development of advanced imaging technologies, specifically computed tomography (CT), enables healthcare providers to detect multiple diagnosis elements, including complete or incomplete bowel blockages with potential strangulation complications [9]. Additionally, ultrasonography has proven valuable in certain acute settings, especially in pediatric or pregnant patients [10]. Despite these investigative tools, a comprehensive clinical assessment remains indispensable, as it often guides the urgency and nature of surgical intervention. Management strategies for intestinal obstruction are highly variable. Conservative approaches include nasogastric decompression, intravenous fluid resuscitation, and correction of electrolyte imbalances. Surgical intervention becomes imperative in the presence of signs indicating strangulation, perforation, or failure of conservative treatment [11]. Laparoscopic approaches have gained popularity due to reduced postoperative complications and faster recovery times [12]. The choice of surgical procedure depends on the etiology, ranging from a dhesiolysis and resection-anastomosis to stoma creation in critically ill patients [13]. Therefore, it is of interest to emphasize the importance of timely intervention, informed surgical decision-making, and diligent postoperative care in reducing morbidity and mortality associated with intestinal obstruction.

## Materials and Methods:

## Study design and setting:

The research was conducted at the General Surgery department of a tertiary care hospital over two consecutive years. The study received institutional ethical approval before the initiation of research. The study obtained written consent from every patient and their guardians when present.

## Study population:

One hundred patients showing clinical indications of intestinal obstruction through abdominal pain together with vomiting, abdominal distension, and obstipation entered the study. Adult patients (aged 18 years or older) who received a diagnosis of acute or subacute intestinal obstruction through clinical examination and radiological results were eligible for admission to the study. The research excluded patients with postoperative ileus who showed no mechanical cause of their symptoms, along with chronic pseudo-obstruction cases, and any patients under 18 years old.

## Data collection:

As patients entered the facility, the doctors obtained extensive patient assessments targeting symptom duration along with existing conditions and previous surgical operations on the abdomen. The physician-focused physical tests evaluate abdominal distension, along with peristaltic sounds and the presence of masses, as well as hernia openings. Medical testing consisted of a complete blood count as well as electrolyte assessment, renal function tests, and arterial blood gas measurements. Plaintext upright abdominal X-rays served as the initial stage of radiological examination, and additional testing involved either ultrasound or CT imaging protocols, depending on the specific clinical case. The appropriate steps included supplying intravenous fluids and performing electrolyte correction together with nasogastric decompression.

## Surgical management and pathological assessment:

Medical staff assessed the need for surgery through a combination of deteriorating clinical health symptoms with imaging verification of the condition alongside unsatisfactory outcomes from 48-72 hours of non-operative care. Medical personnel documented all operative findings to detail which section of the bowels was obstructed, together with the status of bowel viability, and recorded any adhesions, hernias, volvulus tumors, and additional pathologies present. Doctors performed non-viable bowel segment resection with primary anastomosis as tolerated, yet created stomas only for patients who experienced substantial contamination and unstable blood pressure. The removed tissue specimens underwent histopathological analysis to identify exact causal disorders ranging from malignancies to inflammatory diseases.

## Follow-up:

Patients were monitored through the postoperative phase for complications, including surgical site infections, anastomotic leaks, prolonged ileus, and cardiopulmonary issues. Follow-ups continued for six weeks after discharge, with clinical assessments and, when necessary, radiological investigations to evaluate recurrence or complications.

## Results:

## Overall patient characteristics:

A total of 100 patients were included, with a male-to-female ratio of approximately 2:1. The mean age was 51.2 years (range 18-85), with nearly 60% of cases falling into the 40-60 age group. Common presenting complaints included acute onset of colicky abdominal pain (89%), vomiting (78%), and obstipation (66%). About 70% of patients reported a prior history of abdominal or pelvic surgeries, frequently associated with adhesive bands, underscoring the role of postoperative adhesions in causing small bowel obstruction. Most patients arrived at the hospital within 48 hours of symptom onset; however, 20% presented later than 72 hours, often correlating with more severe forms of obstruction and an increased likelihood of strangulation.

## Etiological distribution:

Based on surgical and pathological correlation, adhesions were found to be the most frequent cause of intestinal obstruction (45% of cases). Obstructed external hernias, predominantly incisional and inguinal hernias, accounted for 20% of the cases, followed by malignant strictures and masses (15%), volvulus (10%) and intussusception (5%). Other miscellaneous causes, such as Crohn's strictures and gallstone ileus, contributed to the remaining 5% (Table 1).

## Clinical and radiological findings:

Physical examinations commonly revealed distended abdomens with hyperactive or tinkling bowel sounds on auscultation in early obstruction. Abdominal X-rays were performed in all patients; 65% demonstrated dilated small bowel loops, whereas 25% showed gross colonic distension suggestive of significant bowel obstruction. CT scans were used in 60% of the cohort to delineate the exact site and cause of obstruction precisely; these were instrumental in identifying strangulated segments, volvulus, and subtle adhesions that were not visible on plain X-rays.

## Etiological distribution of intestinal obstruction:

Figure 1 (see PDF) illustrates the distribution of cases by cause of intestinal obstruction.

## Management approaches:

[1] All patients initially underwent conservative management, including nasogastric decompression, fluid resuscitation, and correction of electrolyte imbalances. Definitive surgical intervention was carried out in 80 patients (80%), while the remaining 20% responded favorably to conservative treatment.

[2] Adhesiolysis: Of the 45 patients with adhesive obstruction, 30 underwent adhesiolysis. Fifteen cases were resolved under conservative management. Of those requiring surgery, six required resection and primary anastomosis due to compromised bowel.

[3] Hernia repair: Among patients with obstructed hernias (n = 20), 18 underwent emergent hernia repair with or without mesh placement, while 2 required resection of non-viable bowel segments and subsequent anastomosis.

[4] Oncological resections: Of the 15 malignant cases, 10 underwent resection and primary anastomosis (right hemicolectomy, left hemicolectomy, or segmental resections), while 5 underwent palliative stoma formation due to locally advanced disease or metastatic involvement.

[5] Volvulus management: Sigmoid volvulus (n = 6) was initially managed by endoscopic decompression, where feasible, followed by elective resection and anastomosis. Cecal volvulus (n=4) predominantly required urgent laparotomy and resection of the necrotic bowel segment.

[6] Intussusception: Adult intussusception (n = 5) was often associated with a lead point, such as a polyp or submucosal lesion. Surgical resection was performed in all 5 cases (Table 2).

## Operative and postoperative outcomes:

Intraoperative assessment revealed that 18 patients (18%) had evidence of partial strangulation or compromised bowel, necessitating resection. None of the patients in the conservative group deteriorated significantly to require delayed surgery. Postoperative complications were documented in 30% of operated patients, including wound infection (15%), respiratory infections (7%), prolonged ileus (5%), and anastomotic leak (3%). Overall mortality was 4%, with advanced malignant disease and delayed presentation being the key contributors (Table 3).

## Postoperative complications and mortality:

Figure 2 (see PDF) shows the frequency of each type of postoperative complication and the overall mortality rate.

## Discussion:

Intestinal obstruction remains a leading cause of acute abdominal admissions and a substantial source of morbidity [14, 15]. In our study, adhesive obstruction accounted for nearly half of the cases, a finding consistent with previous reports that highlight postoperative adhesions as a prominent cause of small bowel obstruction [16, 17]. Notably, this underscores the importance of surgical techniques that minimize peritoneal trauma and the potential role of anti-adhesive barriers in high-risk procedures [18, 19]. Our data also point to the prevalence of hernia-related obstruction, specifically in patients who may have delayed elective hernia repair-emphasizing the need for targeted preventive strategies and patient education to reduce the risk of acute strangulation [20, 21]. Malignancy-driven obstruction was observed in 15% of our cohort, in line with global epidemiological data showing an increased incidence of colorectal and other abdominal tumors in older populations [22]. One critical concern in these cases is the dilemma between performing curative resections versus opting for palliative procedures, particularly in the presence of metastatic disease. Our approach was guided by intraoperative findings and overall performance status, aiming to balance oncologic clearance with the immediate need to manage obstruction [23]. In many such situations, a multidisciplinary team approach involving surgical oncologists, gastroenterologists, and palliative care specialists proved pivotal for individualized treatment planning and optimized outcomes [24]. The condition of Volvulus, particularly in the form of sigmoid volvulus, accounts for a significant portion of large bowel obstructions that occur in specific geographic areas due to diet-related factors and natural body configurations [25]. Our medical center benefits from using endoscopic decompression along with definitive surgical treatment to manage sigmoid volvulus. Still, patients with cecal volvulus often require urgent emergency surgery because of their higher chances of tissue death. The correct early detection of volvulus depends strongly on imaging tools, such as abdominal radiography and computed tomography, which demonstrate the importance of rapid radiological assessment [26]. Timely medical care became the key factor in achieving optimal results from treatment. Those who presented within 48 hours generally had fewer complications and more favorable prognoses, whereas delayed presentation often led to strangulated or gangrenous bowel segments requiring extensive resection. Such findings underscore the need to raise public awareness about the seriousness of acute abdominal symptoms and to strengthen emergency healthcare infrastructure for quicker diagnoses and interventions. Indeed, public health campaigns focusing on early recognition and prompt medical evaluation could substantially reduce the burden of advanced disease at presentation [27, 28]. A notable emerging consideration is the role of minimally invasive surgery in managing certain cases of acute intestinal obstruction, particularly those attributable to adhesions. Laparoscopic approaches, when feasible and performed by experienced teams, can reduce postoperative pain, hospital stay, and adhesion formation [29]. However, patient selection and the surgeon's expertise are critical, as complex obstructions or hemodynamic instability often mandate an open approach. Limitations of this study include its relatively small sample size and the fact that it was conducted at a single tertiary institution, potentially limiting its generalizability. Moreover, the lack of a standardized approach to postoperative adhesion prevention among surgeons may have influenced outcomes related to adhesion formation. Future research could benefit from larger, multi-center cohorts and randomized trials investigating prophylactic measures against adhesions, as well as the development of standardized protocols for imaging and intervention. Such efforts would further clarify best practices and refine management pathways for intestinal obstruction, ultimately improving patient outcomes and reducing healthcare costs [30].

## Conclusion:

Intestinal obstruction is a significant and potentially life-threatening condition that demands prompt evaluation and management. Hence, adhesions, hernias, malignancies, and volvulus are the most prevalent causes. Early recognition through a combination of clinical assessment and imaging, along with timely surgical intervention when indicated, is crucial in preventing serious complications.

## Figures and Tables

**Table 1:** Etiological distribution of intestinal obstruction (N=100)

**Etiology**	**Number of Cases (%)**
Adhesions/Bands	45 (45%)
Obstructed Hernias	20 (20%)
Malignancies	15 (15%)
Volvulus	10 (10%)
Intussusception	5 (5%)
Others (Crohn's, etc.)	5 (5%)

**Table 2:** Type of surgical intervention (N=80)

**Surgical Procedure**	**Number of Patients (%)**
Adhesiolysis Only	24 (30%)
Adhesiolysis + Resection + Anastomosis	6 (7.5%)
Hernia Repair (± Resection)	20 (25%)
Resection + Primary Anastomosis (Malignancy)	10 (12.5%)
Palliative Stoma (Malignancy)	5 (6.25%)
Volvulus Correction + Resection	10 (12.5%)
Intussusception Resection	5 (6.25%)

**Table 3:** Postoperative complications and mortality (N=80)

**Complication**	**Incidence (%)**
Wound Infection	12 (15%)
Respiratory Infection	6 (7%)
Prolonged Ileus	4 (5%)
Anastomotic Leak	2 (3%)
Mortality	3 (4%)
